# Identification of Gastric Cancer Biomarkers Using ^1^H Nuclear Magnetic Resonance Spectrometry

**DOI:** 10.1371/journal.pone.0162222

**Published:** 2016-09-09

**Authors:** Gokula Krishnan Ramachandran, Wei Peng Yong, Chen Hua Yeow

**Affiliations:** 1 Department of Biomedical Engineering, National University of Singapore, Singapore, Singapore; 2 Department of Haematology-Oncology, National University Cancer Institute, Singapore (NCIS), Singapore; National Research Council of Italy, ITALY

## Abstract

Existing gastric cancer diagnosing methods were invasive, hence, a reliable non-invasive gastric cancer diagnosing method is needed. As a starting point, we used 1H NMR for identifying gastric cancer biomarkers using a panel of gastric cancer spheroids and normal gastric spheroids. We were able to identify 8 chemical shift biomarkers for gastric cancer spheroids. Our data suggests that the cancerous and non-cancerous spheroids significantly differ in the lipid composition and energy metabolism. These results encourage the translation of these biomarkers into in-vivo gastric cancer detection methodology using MRI-MS.

## Introduction

According to World Health Organization, gastric cancer (0.72 million deaths) ranks third in causing cancer death worldwide in 2012, with the highest incidence in the Asia-Pacific region especially in Japan, gastric cancer is the much more prevalent than other countries. The prognosis of gastric cancer is very poor especially for the one that occurs in the cardiac region. In view of the high global incidence rate, early detection of gastric cancer appears to improve survival rates. Mass screening of gastric cancer in Japan has considerably decreased the mortality rate. Usually, the gastric cancer is often diagnosed at the metastatic stage which is very difficult to treat [1,2]. The gastric cancer incidence is higher in developed countries whereas the mortality is high in developing countries, which is attributed to the lack of early detection, environmental changes, and lifestyle changes. Hence, the economic burden in terms of morbidity and mortality cost will be higher in the developing countries [3]. The gold standard for the diagnosis of gastric cancer is esophagogastroduodenoscopy (EGD) and biopsy. When a doctor identifies an abnormal tissue area through EGD, a biopsy will be performed followed by the routine procedure for the confirmation. Even though this method has high specificity and sensitivity, there are several disadvantages in using endoscopy as the first screening tool; to mention a few, it is an invasive procedure with potential complications such as perforation and aspiration and is not a cost-effective screening tool for most of the countries. Hence, this test is out of reach for most of the people in Asia, Africa, and South America. This method is invasive and creates huge discomfort for the patients especially children and elderly.

One promising non-invasive way of detecting and locating cancer is by employing nuclear magnetic resonance as this method safe as it is non-ionising. Cancer cells contain higher levels of phospholipids than normal cells, which is key for the membrane formation in order to cope up enhanced cell proliferation and signal transduction. The alteration of phospholipid composition also plays a pivotal role in cancer invasion, metastasis, and expression of growth factor receptors [4,5]. Various prior studies concluded that 1H MRS could be used to diagnose cancer and also to monitor responses to the cancer treatment [6–9]. Magnetic resonance imaging and magnetic resonance spectrometry in combination can diagnose of prostate cancer. Studies suggest, combined MRI and MRS could serve as an effective test for the low-risk patients. This study also recommends larger studies for the confirmation of this statement [10]. The lipid composition is significantly altered in the extracts of benign (chronic cholecystitis), intermediate (xanthogranulomatous cholecystitis) and malignant gallbladder tissue. This provides proof that the lipid composition of cancer tissues and normal tissues vary significantly and which can be detected using magnetic resonance [11]. Mobile lipid resonances profiled using 1H MRS can be used to distinguish cervical cancer tissues, low-grade intraepithelial neoplasia, and non-cancerous tissues [12]. This study serves as the proof for the diagnosis of cancer using lipid signals from 1H magnetic resonance. These studies provide an insight that there is a possibility for the presence of a specific mechanism in the cancer cells that alter the phospholipids composition.

*In-vivo* 1H magnetic resonance spectrometry can be used to quantify choline compounds in the breast, it is observed that the choline-containing compounds were significantly higher in malignancies than benign abnormalities and normal breast tissue. Sensitivity, positive predictive value, and accuracy of the cancer diagnosis can be enhanced when 1H MRS and biopsy are employed together. This study also proves that 1H MRS has better sensitivity, positive predictive value, and accuracy that biopsy [13]. 1H MRS is a potential procedure for detecting cancerous breast lesions that are 15mm or larger in diameter without the need of invasive endoscopy procedure [14].

Magnetic resonance method not only identifies lipid biomarkers but also metabolic biomarkers. In human prostate tissues, 1H MRS signals of citrate, creatine, and choline compounds were observed. The citrate to choline ratio can be used for the cancer diagnosis. This ratio is lower for the cancerous tissues in comparison with the noncancerous normal tissues. 1H MRS is a promising method for the detection and treatment follow-up for prostate cancer [15]. Few studies reported that the *in vitro* 1H magnetic resonance spectrometry (MRS) of the perchloric acid extracts of the breast tissue showed resonance at 3.2 ppm, which was significantly high for the breast carcinoma when compared to the normal and benign breast tissue.

The higher levels of the choline related compounds may be primarily attributed to the oncogene-induced activation of phosphatidyl choline and phosphatidyl ethanolamine-specific phospholipases. This resulted in the accumulation of choline related compounds in tumorous or actively proliferating cells [16]. Differences in the concentration of choline-containing compounds can be considered as an indicator for accessing the clinical response of cancer to chemotherapy. The outcome of the chemotherapy can be considered as positive if the choline-containing compounds decrease after the chemotherapy [17].

The biomarkers identified by the 1H-MRS and Nuclear magnetic resonance spectrometry (NMR) could be the same as both these methods employ same principle. Nuclear magnetic resonance spectrometry identifies a unique chemical shift biomarker 1.28 ppm for the identification of the neural stem and progenitor cells (NPC). They could even identify the NPC in the live mouse using this biomarker under proton magnetic resonance spectroscopy (1H-MRS) in a xenografted mice [18]. This is possible as each cell type has unique cell surface receptor proteins and phospholipid composition. From the previous studies, it is clear that magnetic resonance can be employed for cancer diagnosis using the lipid signals.

We hypothesize that 1 dimensional H^1^ Nuclear Magnetic Resonance (NMR) can distinguish between the in-vitro 3D models of the normal gastric cells and gastric cancer cells with reliable NMR chemical shift markers. In this study, we are using the *in-vitro* 3D models (spheroids) of the normal gastric cell line and gastric cancer cell lines to decipher the magnetic resonance based gastric cancer markers. The rationale for using spheroids is that it shows similar characteristics of the *in-vivo* tissues. *In-vivo* cells are interconnected to each other as well as to the extra cellular matrix (ECM). Further *in-vivo* cells shows many characteristic properties like polarization, intercellular communication, developing extracellular matrix and so on. All these unique properties can be replicated in in-vitro spheroids and these are not possible in 2D culture [19,20].

## Methods and Materials

### Cell culture

For this study, a set of twelve gastric cancer cell lines namely SNU484, MKN28, MKN7, SCH, AGS, IST1, KATO3, YCC10, YCC11, N87, NUGC3 and NUGC4 and a normal gastric cancer cell line HS738. The Gastric cancer cells were cultured in RPMI medium with 10% fetal bovine serum (FBS) and the normal gastric cell line was cultured in Dulbecco’s modified essential medium (DMEM) with 10% fetal bovine serum (FBS). All the experiments were carried out in triplicates for all the cell lines along with the spent medium, which serves as background control.

### Formation of cellular spheroids

For the spheroids formation, the cells were grown under 2D culture condition and harvested using trypsinization. The harvested cells were checked for the viability using bromophenol blue dye. The samples with required viability (95%) were diluted into 2.5x10^6^ cells/mL and each sample will contain 1X10^7^ cells I.e., 4 mL of the diluted cell suspension. The diluted cell samples were drawn into drops of 20μL on the lid of the sterile petri dish. The petri dish was filled with 10mL of DPBS for hydrating the drops. The petri dish lid was gently inverted and placed in the petri dish and placed in the incubator for the spheroid formation [21]. The petri dish was then incubated for 3 days. After 3 days, the spheroids could be observed by the naked eye as white spheres. The spheroids were harvested, washed twice with DPBS and gently suspended in 600μL of D_2_O. As a final step, the suspended spheroids were then carefully poured into the NMR tubes.

### Nuclear magnetic resonance (NMR) spectrometry

**Fig 1:**
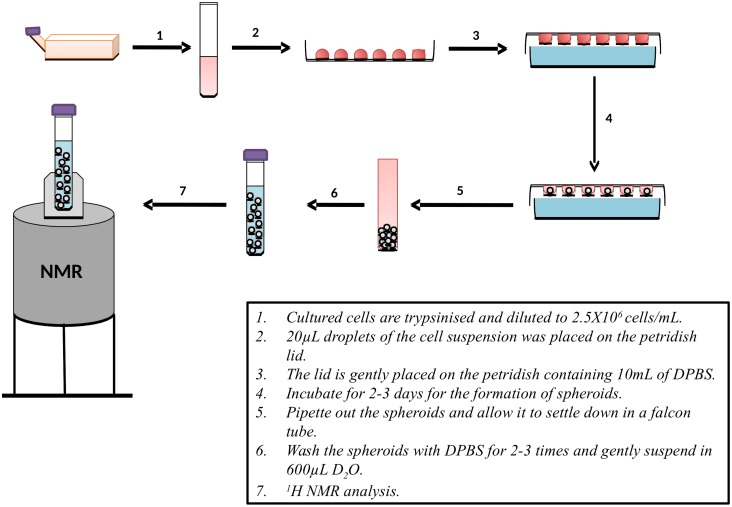
Methodology of sample preparation.

We performed one-dimensional ^1^H NMR spectrometry (DRX500, Bruker USA) on the collected samples and matched controls using deuterated water (D_2_0) we used as the solvent (Fig 1). The acquired NMR spectral data was Fourier-transformed followed by phase and baseline correction using the Bruker XWinNMR software version 3.5. The peak lists were extracted for the metabolite identification. The peak list was given as input to the Metaboanalyst 3.0 online statistical software (www.**metaboanalyst**.ca/, Canada) for statistical analysis [22].

## Results

### Spheroid formation

Among the 12 gastric cancer cell lines, only 4 of them can form the spheroids using the hanging drop procedure mentioned above. The gastric cancer cell lines that can form spheroids were SNU484, NUGC 3, MKN28 and IST1. Among them, the spheroids formed by the SNU484 and MKN 28 were more compact compared to the spheroids formed by IST1 and NUGC 3 cell lines.

### NMR spectrum of gastric cancer and normal gastric spheroids

The peak lists of all NMR spectrums were obtained. The peaks were grouped together by the moving window of 0.03 ppm and a step of 0.015 ppm, which results in a group of 117 peaks. The grouped data was normalized using generalized log transformation and auto scaling.

### Statistical analysis of the NMR spectrums of gastric cancer and normal gastric spheroids

**Fig 2:**
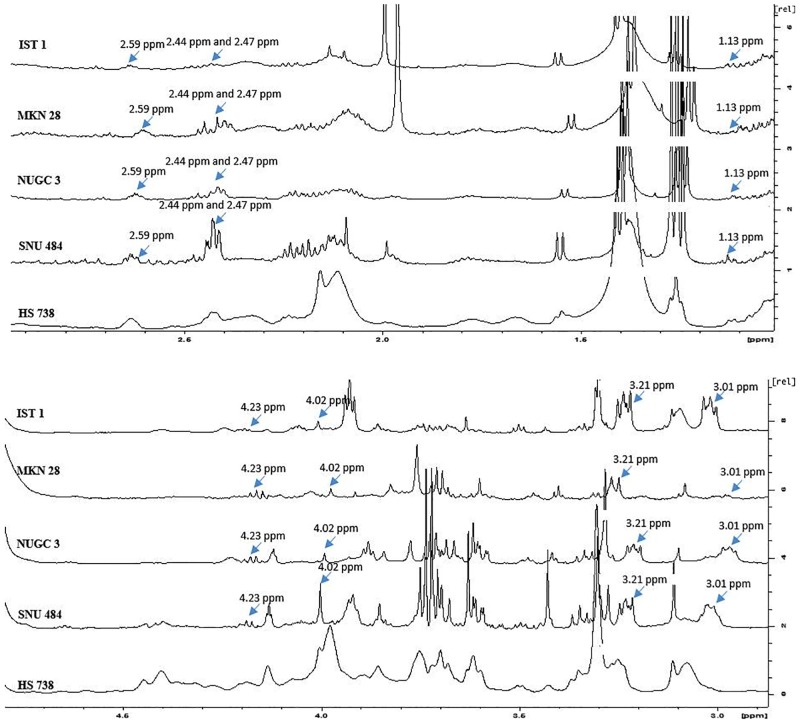
1H NMR spectrum showing NMR markers of gastric cancer spheroids.

**Fig 3:**
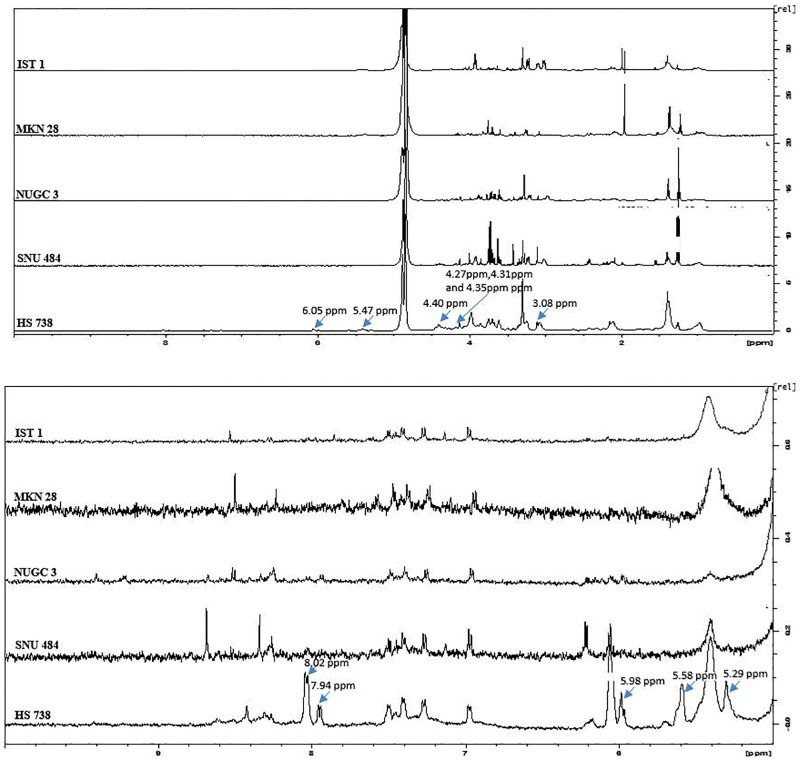
1H NMR spectrum showing NMR markers of normal gastric spheroids.

The statistical analysis the normalized peak lists shows 21 chemical shift peaks (Table 1) were significantly vary between the gastric cancer cell lines (SNU484, MKN28, IST1, and NUGC3) and normal gastric cell line (HS738). Among these 21 chemical shift peaks 8 were present in higher intensity in the gastric cancer spheroids when compared to the gastric normal spheroids. These 8 peaks could be the potential magnetic resonance based gastric cancer markers. The rest 13 were observed to be in higher intensity in the gastric normal spheroids compared to the gastric cancer spheroids. The NMR markers for the gastric cancer spheroids ranges from 1 ppm to 4 ppm (Fig 2) whereas, NMR markers for the normal gastric spheroids ranges from 3 ppm to 8 ppm (Fig 3).

**Table 1:** List of NMR chemical shift markers for the gastric cancer spheroids.

S.No	Chemical shifts (ppm)	FC	log2(FC)*	p.value
1	3.21	332.77	8.3784	0.00021027
2	4.02	53.079	5.7301	0.00044346
3	4.23	44.809	5.4857	0.00096607
4	1.13	34.183	5.0952	0.00092528
5	2.59	16.297	4.0265	0.0067511
6	3.01	15.039	3.9107	0.0085235
7	2.44	7.5685	2.92	0.0012475
8	2.47	4.7224	2.2395	0.0011457
9	4.40	0.17478	-2.5164	0.0002721
10	7.94	0.12923	-2.952	0.00010053
11	3.08	0.12643	-2.9836	0.0088915
12	4.27	0.12609	-2.9874	0.0050839
13	5.29	0.11044	-3.1787	0.00038551
14	5.47	0.10526	-3.248	0.0046374
15	5.98	0.098221	-3.3478	5.67E-06
16	6.05	0.076551	-3.7074	0.00034459
17	4.35	0.05315	-4.2338	0.00074306
18	4.31	0.047047	-4.4098	0.00010888
19	5.58	0.045401	-4.4611	1.30E-11
20	4.72	0.042526	-4.5555	1.48E-16
21	8.02	0.02419	-5.3694	7.49E-12

*positive values represent the chemical shifts exhibit higher intensity in gastric cancer spheroids and negative values represents the chemical shifts exhibit high intensity in normal gastric spheroids.

**Fig 4:**
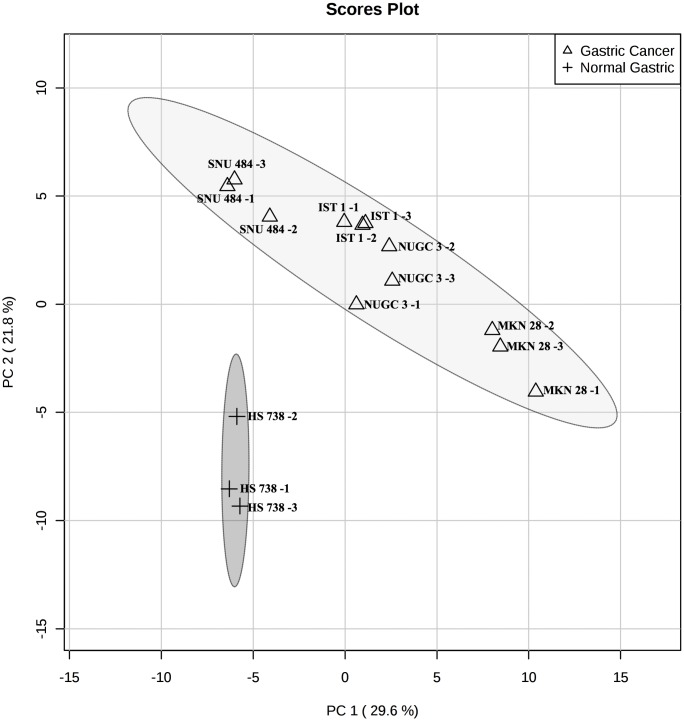
PCA plot depicting the variance between the gastric cancer spheroids and normal gastric spheroids.

**Fig 5:**
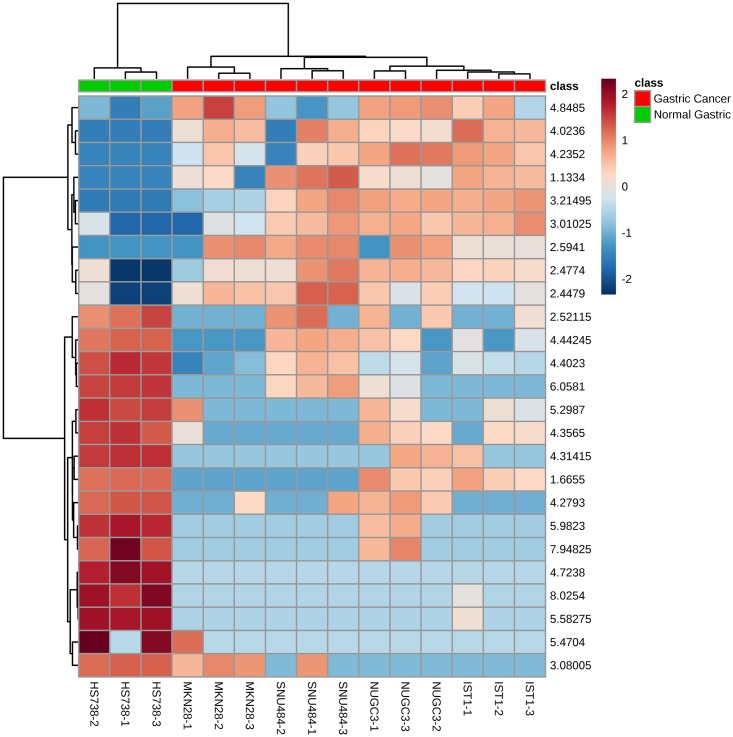
Heat map depicting the relative intensity of the top 25 significant chemical shifts (p value <0.05).

Furthermore, principal component analysis (PCA) and clustering analysis performed between gastric cancer and normal gastric spheroids depict the gastric cancer spheroids and normal gastric spheroids into different clusters, indicating the significant variance between these cell lines (Fig 4). Heat map analysis manifests the top 25 significant (p-value <0.05) chemical shift markers with its relative intensities (Fig 5).

## Discussion

### Formation of gastric spheroids

In this study, we have observed that spontaneous spheroid formation is not common for all the cell lines only a few cell lines like IST 1, NUGC 3, MKN 28, SNU 484 and HS 738 can form spontaneous spheroids. This is because cells interact with each other in a very complex and diverse manner which dictates the formation of the spontaneous spheroids *in-vitro*. In a previous study which involves 8 breast cancer cell lines show that the E- Cadherin plays a pivotal role in the formation of spontaneous spheroids in some cell lines whereas the interaction between the collagen I/integrin ß1 drives the cells to form spontaneous spheroids. Interestingly some cell lines were able to form spheroids with the shared responsibility of both homophilic E-cadherin and integrin ß1/collagen I [23].

### Phospholipid metabolism is the major contributor of NMR markers for Gastric cancer

We could observe a total of eight NMR markers that are overexpressed in gastric cancer spheroids than normal gastric spheroids. 3.2 ppm represents the choline and choline related compounds [24]. Choline has been identified as the tumor marker in breast cancer and lung cancer [13, 25]. Choline and its derivatives have been already reported as the markers of cell proliferation as they play a key role in phospholipid metabolism that forms the cell membrane [26]. The chemical shift 1.13 ppm has been already identified as the marker for cervical cancer. The serum obtained from the cervical cancer patients shows 1.13 ppm as the marker for the diagnosis of cervical cancer [27].

Our study identifies chemical shift at 3.01 ppm as a marker for gastric cancer. This chemical shift represents creatine [28,29]. Creatine was reported to the biomarker for cervical cancer. Ex-vivo HR-MRS analysis of the cervical tumor tissue and normal cervical tissue shows a significant (p = 0.004) difference in creatine [30]. Creatine is named as a potential biomarker for lung cancer in a study which involves analyzing the methanol-chloroform—water extract of non-small cell lung cancer tissues from 21 lung cancer patients [25]. This supports our data which shows creatine, represented by the chemical shift at 3.01 ppm as the marker for gastric cancer. Another important gastric cancer biomarker that we have discovered is the chemical shift at 4.02 ppm, which represents 2 hydroxyglutarates. World Health Organization reported that 2 hydroxyglutarate is the key biomarker for the identification of IDH (Isocitrate dehydrogenase) mutated glioma as the mutation in these genes could result in the accumulation of the 2-hydroxyglutarate [31]. In our knowledge, we are the first to report 4.02 ppm as the biomarker for gastric cancer.

Another interesting chemical shift marker for gastric cancer is the chemical shift at 2.47 ppm. This marker indicates glutamine, a common amino acid. Previous studies have reported glutamine as the marker for the slightly malignant prostate cancer [32]. Our study depicts glutamine as the common NMR marker for gastric cancer as well as prostate cancer. The chemical shift marker 2.59 ppm is assigned to methylamine. Methylamine has been reported, as the key biomarker is cancers of the alimentary canal. Methylamine, as represented as the chemical shift 2.59 ppm, has been reported as the rectal cancer marker. Methylamine involves in the metabolism of another important cancer biomarker namely choline [26]. It is reported that methylamines can cause hepatocarcinogenesis in rats [33]. It is also reported that higher levels of methylamine could induce gastric cancer [34]. 2- oxoglutarate plays a pivotal role in the activity of the 2- oxoglutarate-dependent dioxygenases. Increase in the cellular level of 2- oxoglutarate elevates the activity of the 2- oxoglutarate-dependent dioxygenases, which will result in many crucial events like activation of hypoxia inducible factors (HIF 1), disturbances in the epigenetic regulation and reduction of the carnitine synthesis. These events eventually lead to tumor viability and metastasis [35]. Our study depicts the presence chemical shift marker for gastric cancer at 2.44 ppm which is assigned to 2 –oxoglutarate [36].

### Glycosylated derivatives are the key NMR markers unique to the normal gastric spheroids

We observed 13 NMR chemical shift markers that are unique to the normal gastric spheroid that is not found or varies significantly (p<0.05) from the gastric cancer spheroids. The assignment of these markers and their role were as follows: The chemical shifts at 4.40 ppm and 4.27 ppm indicate the oligosaccharide alditols, these compounds consist of the Galβ1-3(GlcNAcβ1–6)- GalNAc-ol core. This core contains the O-glycosidic structures. These structures are represented by the chemical shifts of 4.40 ppm and 4.27 ppm [37,38].

Our data depicts the strong evidence for the presence of UDP-glucose. Chemical shift resonance at 8.02 ppm represents the proton in the NH group of the uracil ring [39,40], whereas the NMR resonances at 7.94 ppm and 5.98 ppm mark the presence of the protons linked to the C6 and C5 of the uracil ring [41]. The presence of the phosphate group is indicated by the chemical shift resonance at 4.35 ppm [42]. This evidence proves the existence of the uridine diphosphate in the normal gastric spheroids. There must be a glucose molecule which is hinted by the chemical shift resonance at 4.27 ppm and that is linked with the UDP via anomeric carbon as we also observed a chemical shift resonance at 5.58 ppm which manifests the occupancy of the proton-linked with the anomeric carbon of glucose [43].

It is reported that increase in the synthesis of glycogen from glucose results in the decrease in the cellular concentration of the UDP-glucose [44]. On the other hand, the increased glycogen synthesis has been demonstrated in many types of cancer [45]. These studies support our data, which shows a significant increase in the UDP-glucose in the normal gastric spheroids compared to the gastric cancer spheroids. The chemical shift at 4.72 ppm and 4.31 ppm implies β (1–4) N-acetyl galactosamine, where the 4.72 ppm and 4.31 ppm denotes H-1β [46] and β(1–4)GalNAc respectively [47,48]. 4.31 ppm precisely indicates galactosamine as well as the acetylation of galactosamine in N-acetyl galactosamine [47,49]. Both 4.72 ppm, as well as the 4.31ppm, reveals the presence of β (1–4) configuration [46,48].

Chemical shift at 5.47 points out the H linked to the C_1_ of the N-acetyl galactosamine of UDP-GalNAc [50]. In another study, it also denotes the existence of a-D-GlcpNAc [51]. another study describes this chemical shift for H-1 of α-Gal-1-P [52]. These observations strongly suggest the presence of UDP-GalNAc as we already have evidence for the presence of UDP. The chemical shift at 5.29 ppm manifests the proton from anomeric carbon [53] and specifically at β anomeric configuration [54]. Prior study has reported that this chemical shift indicates the galactopyranose residue at the reducing end of glycolipids [53]. Another report which characterizes the liver fats shows that 5.29 ppm belongs to the olefinic group in the triglycerides [55]. All this data suggests that the gastric cancer spheroids and normal gastric spheroids vary significantly in the composition of lipid and lipid derivatives. Another chemical shift marker which is significantly higher in normal gastric spheroids, 6.05 ppm is assigned to NH_2_ group in a prior study [56,57]. Interestingly another report had assigned this chemical shift to the protons from the olefinic group [58,59].

Finally, the chemical shift at 3.08 is attributed to proton from the CH_2_ group of the methyl lysine. Although it is previously reported that this chemical shift manifests the presence of the SH_2_ group in taurine [60]. Another study where proton nuclear magnetic resonance spectrometry is utilized for the identification of the neural cell types supports the assignment of 3.08 ppm to the SH_2_ of taurine [61]. In these studies, the 3.08 ppm chemical shift has appeared as triplet but in our data, the 3.08 ppm appear as a singlet. In a previous report, where the chromatin core particles where analyzed using proton nuclear magnetic resonance spectrometry encountered the 3.08 ppm chemical shift as singlet and it was assigned to the CH_2_ group proton of the methyl lysine [62]. Methylated amino acids especially methyl-lysine and methylarginine in the chromatin plays a vital role in recruiting proteins that induce structural changes in chromatin thus influencing gene expression and repression [63]. Our data suggests that the gastric cancer spheroids have significantly lesser methylated amino acids thus impairing its epigenetic control over gene expression.

### Feasibility of clinical translation of this study

In this study we used the 3D spheroids, in order to minimize the limitations in its clinical translation. Flat monolayer cultures are the simplistic cancer models, where the cells are adhered to the poly D- Lysine treated plastic surfaces or glass surfaces. These cancer models have physiologically uniform environment and lacks cell to cell attachment, which contradicts the actual tumor environment. In *in vivo* tumors there exists cell to cell attachment, oxygen gradient, nutrition gradient and waste gradient. This *in vivo* tumor environment could be reproduced in the 3D spheroids with ease. Generally solid tumors proliferate in a differential manner whereas the proliferation is higher at the periphery than the core region which is attributed to the oxygen and nutrient gradient. This behavior could accurately be depicted by the 3D spheroids whereas, cells grown in 2D flat monolayer depicts uniform proliferation [64]. 2D flat monolayer cultured cells shows apical-basal polarity as only one side of the cell is adhered to the surface and that has a huge impact on the cellular function. As these 2D cultured cells always depicts simple geometry they don’t show histological differentiation of the *in vivo* tumors but by just growing cells as 3D spheroids one could obtain the histological morphology similar to that of the *in vivo* tumor type from which the cell lines were derived [65, 66]. The cells in a tumor exhibits phenotypic heterogeneity in the cell proliferation rate, gene expression and differentiation which leads to the heterogeneity in the function and morphology [67]. Tumor spheroids could capture this phenotypic heterogeneity as they have oxygen and nutrient gradients. Few cells exhibit the stem cell like characteristics such as self-renewal and undifferentiated multipotent phenotype called cancer stem cells (CSCs) [68]. These CSCs are observed in both *in vitro* 3D tumor spheroids and *in vivo* tumors [69] and these stemness-related genes are found to be upregulated in 3D spheroids compared to the 2D monolayers [70]. The gene expressions profiles including the expression of transcription factors of a cell line grown as 3D spheroids is divergent compared to the same cell line cultured 2D monolayers [66, 71, 72]. All the above studies show the cells grown as 3D spheroids resemble closely to the cells in the *in vivo* tumors. Thus, we expect there won’t be any potential limitations in the clinical translation of the results of this study and of course an actual clinical translational study is necessary to corroborate this statement.

## Conclusion

In this study, we decipher the NMR markers for differentiating gastric cancer spheroids and normal gastric spheroids. We are able to identify 8 markers that are unique to the gastric cancer spheroids that are analyzed in this study. We also demonstrated that there are 13 markers that are significantly lesser in the gastric cancer spheroids compared to their normal counterpart. These markers indicate that the cancerous and non-cancerous spheroids differ majorly in the energy metabolism, composition of lipid and lipid derivatives. This opens up avenues for the researches focusing on identifying novel lipid targets in gastric cancer. This study also depicts that the formation of spontaneous spheroids is not a common trait for all the cell lines.
